# Cell-growth phase-dependent promoter replacement approach for improved poly(lactate-*co*-3-hydroxybutyrate) production in *Escherichia coli*

**DOI:** 10.1186/s12934-023-02143-w

**Published:** 2023-07-19

**Authors:** Yuki Nagao, Sangho Koh, Seiichi Taguchi, Tomohiro Shimada

**Affiliations:** 1 School of Agriculture, Meiji University, 1-1-1 Kawasaki-Shi, Kanagawa, 214-8571 Japan; 2 Graduate School of Science, Technology and Innovation, Kobe University, 1-1 Rokkodai-Cho, Nada, Kobe, 657-8501 Japan; 3 Engineering Biology Research Center, Kobe University, 1-1 Rokkodai-Cho, Nada, Kobe, 657-8501 Japan

**Keywords:** P(LA-*co*-3HB), Promoter, Gene expression, Lactate fraction, *Escherichia coli*

## Abstract

*Escherichia coli* is a useful platform for producing valuable materials through the implementation of synthetic gene(s) derived from other organisms. The production of lactate (LA)-based polyester poly[LA-*co*-3-hydroxybutyrate (3HB)] was carried out in *E. coli* using a set of five other species-derived genes: *Pseudomonas* sp. 61-3-derived *phaC1STQK* (for polymerization), *Cupriavidus necator-*derived *phaAB* (for 3HB-CoA generation)*, and Megasphaera elsdenii-*derived *pct* (for LA-CoA generation) cloned into pTV118N*pctphaC1*p_s_(ST/QK)*AB.* Here, we aimed to optimize the expression level and timing of these genes to improve the production of P(LA-*co*-3HB) and to manipulate the LA fraction by replacing the promoters with various promoters in *E. coli*. Evaluation of the effects of 21 promoter replacement plasmids revealed that the *phaC1STQK-AB* operon is critical for the stationary phase for P(LA-*co*-3HB) production. Interestingly, the effects of the promoters depended on the composition of the medium. In glucose-supplemented LB medium, the *dps* promoter replacement plasmid resulted in the greatest effect, increasing the accumulation to 8.8 g/L and an LA fraction of 14.1 mol% of P(LA-*co*-3HB), compared to 2.7 g/L and 8.1 mol% with the original plasmid. In xylose-supplemented LB medium, the *yliH* promoter replacement plasmid resulted in the greatest effect, with production of 5.6 g/L and an LA fraction of 40.2 mol% compared to 3.6 g/L and 22.6 mol% with the original plasmid. These results suggest that the selection of an appropriate promoter for expression of the *phaC1STQK-AB* operon could improve the production and LA fraction of P(LA-*co*-3HB). Here, we propose that the selection of cell-growth phase-dependent promoters is a versatile biotechnological strategy for effective intracellular production of polymeric materials such as P(LA-*co*-3HB), in combination with the selection of sugar-based carbon sources.

**Supplementary Information:**

The online version contains supplementary material available at 10.1186/s12934-023-02143-w.

## Introduction

**Fig. 1:**
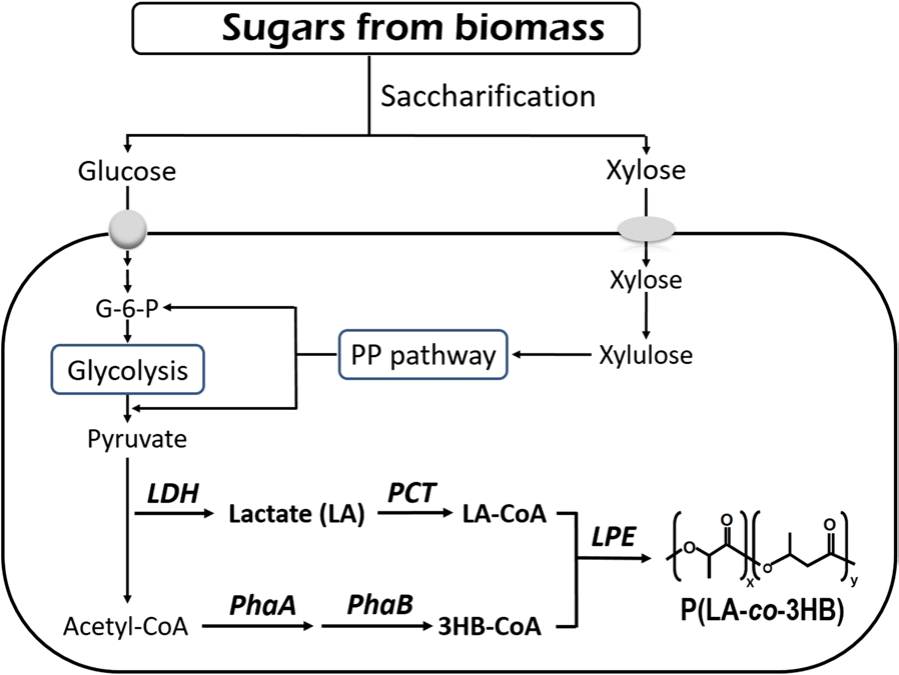
Biosynthetic pathway scheme of P(LA-*co*-3HB) production in *E. coli* used in this study. Two sugars, glucose and xylose, derived from biomass were used as carbon sources for production of P(LA-*co*-3HB) in *Escherichia coli*. LDH: lactate dehydrogenase; PCT: propionyl-CoA transferase; LPE: lactate-polymerizing enzyme [29] engineered from PHA synthase of *Pseudomonas* sp. 61-3; PhaA: β-ketothiolase; PhaB: NADPH-dependent acetoacetyl-CoA reductase

Plastics derived from renewable biomass are of interest because they are a potential solution to dwindling and increasingly expensive petroleum resources and have the capacity to reduce carbon dioxide emissions. Polylactide (PLA), a representative bio-based plastic, can be chemically synthesized in multiple steps from renewable carbon sources. PLA has a wide range of applications, such as biomedical materials and food-related items. However, the stiffness and brittleness of PLA limit its competitive market expansion. In contrast, P(lactate-*co*-3-hydroxybutyrate) [P(LA-*co*-3HB)], an LA-based polyester copolymerized with 3HB, which is a typical constituent of polyhydroxyalkanoates (PHAs), can be processed into flexible and transparent plastic materials and can be used in a broad range of applications (Fig. 1) [29, 30]. Such copolymers are known to exhibit various mechanical and thermal properties depending on their monomer fraction, and thus can be used in a wide range of applications [24, 28]. In addition, P(LA-*co*-3HB) is likely to have certain beneficial properties performed by both homopolymers, such as the high glass transition temperature of PLA and the excellent biodegradability of P(3HB) [23, 30]. Especially, even the highly LA-enriched P(LA-*co*-3HB) exhibits excellent biodegradability in soil environments, and its biodegradation mechanism has been investigated from the viewpoint of enzymes and polymer products [4, 25, 26].

In 2008, Taguchi et al. succeeded in creating a microbial biosynthetic system for P(LA-*co*-3HB) using *Escherichia coli* as a platform (Fig. 1) [29]. In this system, the discovery of an “LA-polymerizing enzyme (LPE),” which is a mutant of PHA synthase PhaC1S325T/Q481K(PhaC1STQK), originally from *Pseudomonas* sp. 61-3, enabled us to construct a one-step biosynthetic system for LA-based polyesters under mild conditions. P(LA-*co*-3HB) is intracellularly synthesized by the following successive enzymatic reaction steps: (i) generation of lactyl-coenzyme A (LA-CoA) by propionyl-CoA transferase (PCT) derived from *Megasphaera elsdenii*, (ii) supply of 3-hydroxybutyryl-CoA (3HB-CoA) via the dimerization pathway (PhaA and PhaB) derived from *Cupriavidus necator*, and (iii) copolymerization of the CoA esters by the LPE. Currently, these sets of genes for the P(LA-*co*-3HB) production are cloned into a plasmid called ‘pTV118N*pctphaC1*p_s_(ST/QK)*AB*’, and *E. coli* transformants are used. With this plasmid, the *phaC1STQK-phaA-phaB* operon is driven by the *phaC* promoter of *C. necator*, and *pct* is driven by the *lac* promoter of *E. coli*.

As described above, P(LA-*co*-3HB) is a copolymer that can have a wide range of properties owing to the LA fraction, which must be produced by an enzymatic reaction for the polymerization of both LA and 3HB, and there is a need to improve not only the production volume but also alteration of the LA fraction. The following various efforts have been made regarding P(LA-*co*-3HB) production in *E. coli* as a platform; the nature of the carbon source of the medium [5, 14, 16, 17, 21, 27], enzymatic modification of enzymes involved in P(LA-*co*-3HB) synthesis [30], deletion or overexpression of carbon source metabolism genes and sugar transporter genes [15, 31], modification of the transcriptional regulatory network by deletion of transcriptional regulators [9, 10, 12], and cell membrane flexibility [11].

In addition, we have accumulated extensive fundamental findings related to the transcription network governing global gene expression in *E. coli* [7]*.* In particular, we have developed a Genomic SELEX system to elucidate the genomic regulatory networks of transcription factors based on the identification of the binding sites in vitro of the test transcription factor on the *E. coli* genome [20]. These long-term studies are logically applicable to the microbial production of value-added products. In general, cell growth phase-separated regulation is appropriate for efficient production of the target product of interest. In particular, caution should be exercised with intracellular biosynthesis of polymeric materials, such as hydrophobic PHA polymers, to avoid cell growth inhibition caused by artificial compound production [3]. Recently, it was reported that membrane vesicles are produced by envelope stress caused by the accumulation of PHB [13]. However, the selection of appropriate promoters for efficient PHA production has not been reported to date.

In the present study, we investigated the effectiveness of promoter selection for increased productivity and altered LA fraction of P(LA-*co*-3HB), considering the cell growth phase of the recombinant *E. coli* strain used. In general, the intercellular synthesis rate of a target compound is affected not only by the activity of the enzyme itself but also by its expression level. Although genetic modifications of *E. coli* as a host strain, carbon sources, and culture conditions have been studied, it might be argued that the plasmid pTV118N*pctphaC1*p_s_(ST/QK)*AB* has been conventionally used for P(LA-*co*-3HB) production. First, we investigated the expression status of the P(LA-*co*-3HB)-synthesizing genes cloned into the plasmid, and we then attempted to alter P(LA-*co*-3HB) production and the LA fraction by optimizing the expression system through promoter replacement of a set of genes involved in P(LA-*co*-3HB) synthesis in the plasmid. As expected, improved production of P(LA-*co*-3HB) and altered LA fraction were achieved using a promoter replacement-based approach in combination with the selection of sugar-based carbon sources.

## Results and discussion

### Correlation of P(LA-*co*-3HB) production pattern with *phaC1STQK-AB* expression pattern by use of the conventional plasmid pTV118N*pctphaC1*p_s_(ST/QK)*AB*

P(LA-*co*-3HB) was produced by culturing *E. coli* harboring the conventional pTV118N*pctphaC1*p_s_(ST/QK)*AB* plasmid in glucose-supplemented LB medium. To determine the adequate amount of glucose in the medium for P(LA-*co*-3HB) production, the production volume and LA fraction of P(LA-*co*-3HB) by pTV118N*pctphaC1*p_s_(ST/QK)*AB* transformants in LB medium with various glucose concentrations were measured. It was found that a glucose concentration of 3% (w/v) was most suitable for P(LA-*co*-3HB) production (Additional file 1: Fig. S1), and a sugar concentration of 3% was fixed in the subsequent experiments.

**Fig. 2:**
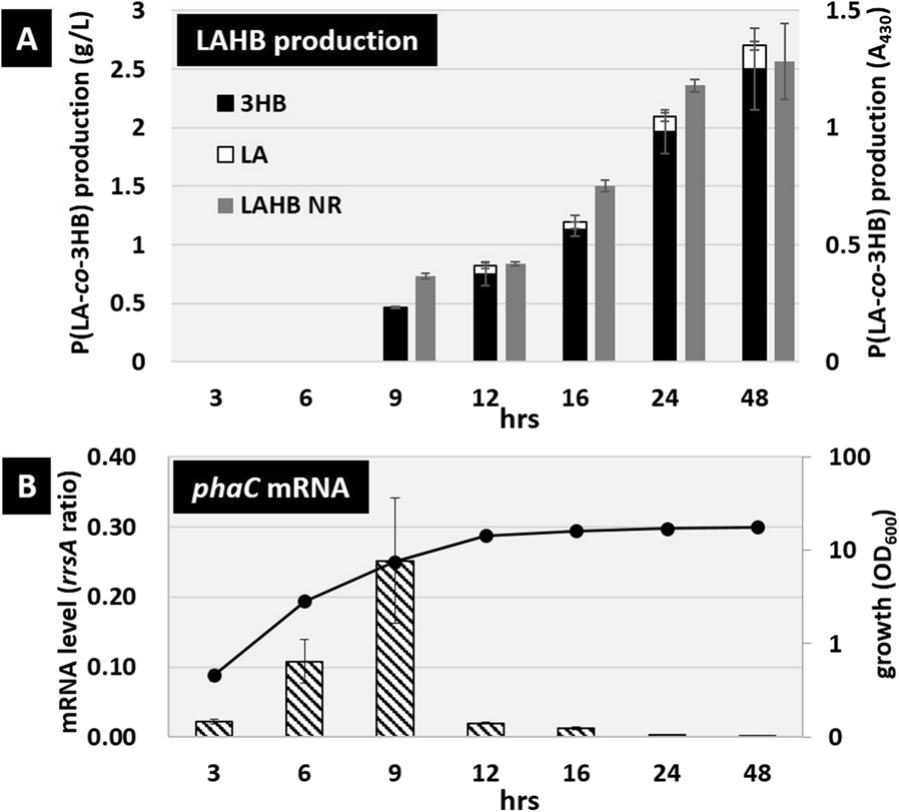
P(LA-*co*-3HB) production and expression levels of *phaC1STQK-AB* operon over the course of cultivation time by *E. coli* harboring pTV118N*pctphaC1*p_s_(ST/QK)*AB* in glucose-supplemented LB medium. **A** The bars indicate the amount of 3HB units in the polymer (black), the amount of LA units in the polymer (white), and the fluorescent intensities of Nile-red-stained cells (gray). **B** Diagonal stripe bars indicate the mRNA levels of *phaC*. The line graph shows cell growth

The production and LA fraction of P(LA-*co*-3HB) in *E. coli* harboring pTV118N*pctphaC1*p_s_(ST/QK)*AB* under these conditions were measured over 48 h. P(LA-*co*-3HB) was measured by two methods: GC-FID was used to quantify the P(LA-*co*-3HB) copolymer in terms of the amount of 3HB and LA in dried cell weight %, and Nile-red staining was used as a semi-quantative measure of the total amount of P(LA-*co*-3HB). From the GC-FID results, accumulation of P(LA-*co*-3HB) was observed after 9 h of cultivation, followed by a gradual increase, reaching 2.7 g/L and an LA fraction of approximately 7.4 mol% after 48 h (Fig. 2A). This result was consistent with the quantification by Nile-red staining. Accumulation of P(LA-*co*-3HB) did not change with further incubation (data not shown). Next, the cell density under culture conditions was measured, and cell growth started to slow at 9 h and transitioned to the stationary phase by 12 h after inoculation (Fig. 2B). These results suggest that, under these conditions, the production of P(LA-*co*-3HB) begins with the transition of cells into the stationary phase.

In contrast, the mRNA expression of the *phaC1STQK-AB* operon responsible for P(LA-*co*-3HB) production was measured under the same conditions and was found to increase from the exponential growth phase to the early stationary phase, followed by a sharp decrease (Fig. 2B). This result indicates that the *phaC* promoter of *C. necator* functions as an exponential phase-dependent promoter in *E. coli* that is active during the exponential phase and inactive during the stationary phase. These results suggest that the enzymes involved in P(LA-*co*-3HB) synthesis expressed in the exponential phase contribute little to P(LA-*co*-3HB) production during the exponential phase. It is also suggested that stationary phase P(LA-co-3HB) production, which is responsible for the main production of P(LA-co-3HB) accumulation, may be carried out by enzymes expressed in the exponential phase by an exponential phase-dependent promoter. This imbalanced correlation between the promoter-mediated gene expression responsible for polymer synthesis and the P(LA-*co*-3HB) production phase was clearly identified as an issue in need of further improvement.

### Effect of promoter replacement of the *phaC1STQK-AB* operon for 3HB-CoA synthesis and copolymerization

**Fig. 3:**
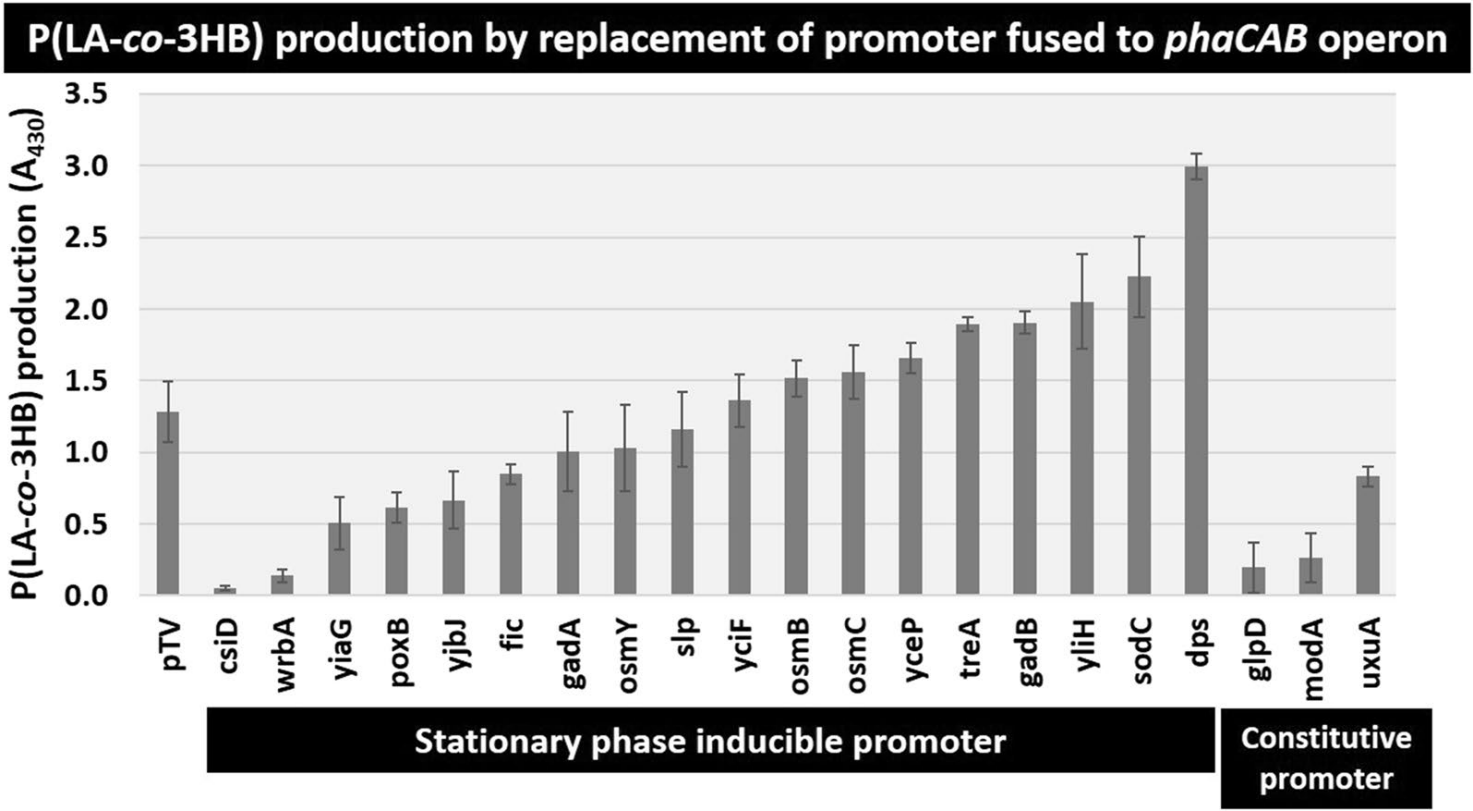
P(LA-*co*-3HB) production by replacement of the promoter fused to the *phaC1STQK-AB* operon. Each P(LA-*co*-3HB) production was measured by the fluorescent intensities of Nile-red staining. pTV indicates the conventional plasmid pTV118N*pctphaC1*p_s_(ST/QK)*AB.* The gene name indicates the promoter replaced with the *phaC* promoter

Since the production of P(LA-*co*-3HB) occurs mostly after the transition to the stationary phase, it was thought that the expression of genes involved in the production of P(LA-*co*-3HB) should also transition into the stationary phase. Previously, we comprehensively measured the activities and properties of *E. coli* stationary-phase promoter by cloning it into a multi-copy plasmid vector and fusing it to a *gfp* reporter gene [18]. Based on these results, we selected 18 stationary-phase-inducible promoters, including those with weak to strong activities, and three constitutive promoters for comparison, and we constructed 21 promoter replacement plasmids instead of the existing *phaC* promoter in pTV118N*pctphaC1*p_s_(ST/QK)*AB*. Each of these plasmids was transformed into the *E. coli* BW25113 strain, and the effect of the promoter replacement on P(LA-*co*-3HB) accumulation was assessed by Nile-red staining of cells 48 h after inoculation (Fig. 3, Table 1). Different amounts of P(LA-*co*-3HB) accumulation were observed for each promoter, and a 1.5- to 2.3-fold increase in production was observed for the stationary-phase-inducible promoters *dps*, *sodC*, *yliH*, *gadB*, and *treA* relative to the original *phaC* promoter. All these promoters have been reported to be dependent on the stationary-phase sigma factor RpoS, which recognizes stationary-phase promoters [18]. In contrast, for the constitutive promoters *glpD*, *modA*, and *uxuA*, which have been shown to exhibit strong constitutive activity in previous reports, the production of P(LA-*co*-3HB) decreased below that of the original *phaC* promoter.

**Table 1:** Effect of replacement of the promoter fused to the *phaC1STQK-AB* operon in pTV118N*pctphaC1*p_s_(ST/QK)*AB* plasmid on P(LA-*co*-3HB) production

Promoter	P(LA-co-3HB) quantification method	Glucose-supplemented LB medium													Xylose-supplemented LB medium									
		Nile red staining			GC-FID																			
	Gene function	A_430_	S.D.	Ratio	3HB (g/L)	S.D.	Ratio	LA (g/L)	S.D.	Ratio	Total (g/L)	Ratio	LA composition (%)	S.D.	3HB (g/L)	S.D.	Ratio	LA (g/L)	S.D.	Ratio	Total (g/L)	Ratio	LA composition (%)	S.D.
Original promoter																								
*phaC *	PHA synthase	1.28	0.21	1.00	2.50	0.35	1.00	0.20	0.04	1.00	2.70	1.00	8	0.00	2.82	0.23	1.00	0.82	0.03	1.00	3.64	1.00	23	0.01
Stationary phase inducible promoter																								
*dps*	Stationary phase nucleoid protein	2.99	0.09	2.33	7.66	0.36	3.06	1.12	0.18	5.62	8.78	3.25	14	0.03	2.55	0.11	0.90	0.82	0.11	0.99	3.37	0.92	24	0.02
*sodC*	Superoxide dismutase	2.23	0.28	1.74	5.16	0.92	2.06	0.56	0.19	2.82	5.72	2.12	12	0.02	2.17	0.58	0.77	0.43	0.05	0.53	2.61	0.72	17	0.01
*yliH*	Regulator of biofilm formation	2.05	0.33	1.60	5.75	0.49	2.30	0.75	0.08	3.77	6.50	2.41	13	0.01	3.34	0.37	1.19	2.25	0.28	2.74	5.59	1.54	40	0.02
*gadB*	Glutamate decarboxylase B	1.91	0.08	1.49	5.27	0.03	2.11	0.35	0.04	1.74	5.61	2.08	8	0.01	3.37	0.26	1.19	1.03	0.03	1.25	4.39	1.21	23	0.02
*treA*	Trehalase	1.89	0.05	1.48																				
*yceP*	Regulator of biofilm formation	1.66	0.11	1.29																				
*osmC*	Osmotically inducible peroxiredoxin	1.56	0.18	1.22																				
*osmB*	Osmotically inducible lipoprotein	1.52	0.13	1.18																				
*yciF*	Osmotically inducible protein	1.36	0.18	1.06																				
*slp*	Starvation lipoprotein	1.16	0.26	0.91																				
*osmY*	Chaperone	1.03	0.30	0.80																				
*gadA*	Glutamate decarboxylase A	1.01	0.27	0.78																				
*fic*	Adenosine monophosphate transferase	0.85	0.07	0.66																				
*yjbJ*	Stress response protein	0.67	0.20	0.52																				
*poxB*	Pyruvate oxidase	0.61	0.11	0.48																				
*yiaG*	Putative transcriptional regulator	0.51	0.18	0.39																				
*wrbA*	quinone oxidoreductase	0.14	0.05	0.11																				
*csiD*	Carbon starvation inducible protein	0.05	0.02	0.04																				
Constitutive promoter																								
*uxuA*	d-Mannonate dehydratase	0.83	0.07	0.65																				
*modA*	Molybdate transporter	0.27	0.17	0.21																				
*glpD*	Glycerol 3-phosphate dehydrogenase	0.20	0.17	0.15																				

**Fig. 4:**
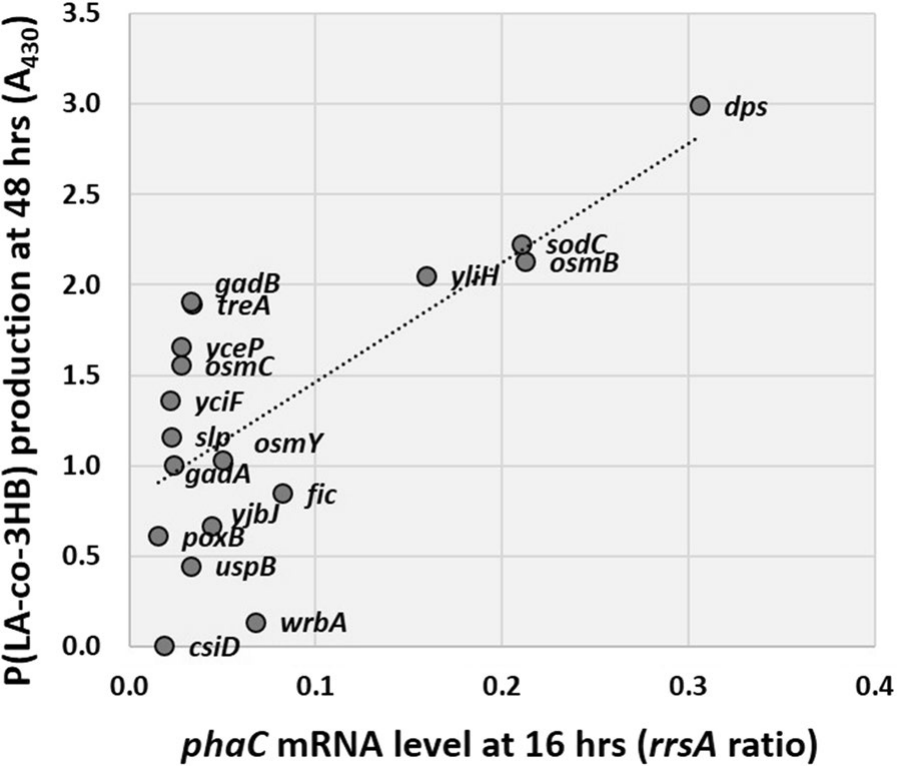
Correlation between P(LA-*co*-3HB) production and the mRNA levels of *phaC.* The x-axis shows the expression ratio of *phaC* mRNA to the ribosomal RNA, *rrsA*, quantified by RT-qPCR after 16 h of incubation. The y-axis shows the production of P(LA-*co*-3HB) as measured by Nile-red staining of cells after 48 h of incubation. The gene name of the promoter used for expression of the *phaC1STQK-AB* operon is indicated next to each dot

To confirm the correlation between the activity of these 18 stationary-phase-inducible promoters and the P(LA-*co*-3HB) accumulation amount, the mRNA expression levels of *phaC* expressed by each promoter at different incubation times (9, 12, 16, and 24 h) and P(LA-*co*-3HB) accumulation were compared. The results show that the expression level at 16 h post-incubation correlated best with the P(LA-*co*-3HB) accumulation (correlation coefficient = 0.700) and was lower at 9 h (0.454) (Fig. 4 and Additional file 1: Fig. S2). To determine the exact amount and LA fraction of P(LA-*co*-3HB) products, copolymers accumulated by promoter replacement plasmids with significantly increased production were measured. We obtained results showing > twofold increase in production relative to the original *phaC* promoter, with the *dps* promoter showing > threefold increase to 8.8 g/L (Table 1, Additional file 1: Fig. S3). In accordance with the effect of this promoter activity optimization, a suitable glucose concentration was re-examined using the *yliH* promoter as a representative example, and 3% glucose was found to be optimal (Additional file 1: Fig. S4). Replacing the *phaC* promoter upstream of the *phaC1STQK-AB* operon with a stationary-phase-inducible promoter successfully increased P(LA-*co*-3HB) production but had little effect on the LA fraction, which was approximately 12 mol% as opposed to 8 mol% for the original promoter (Table 1, Additional file 1: Fig. S3).

### Effect of promoter replacement of *pct* for LA-CoA synthesis and *ldhA* for l-lactate synthesis

**Fig. 5:**
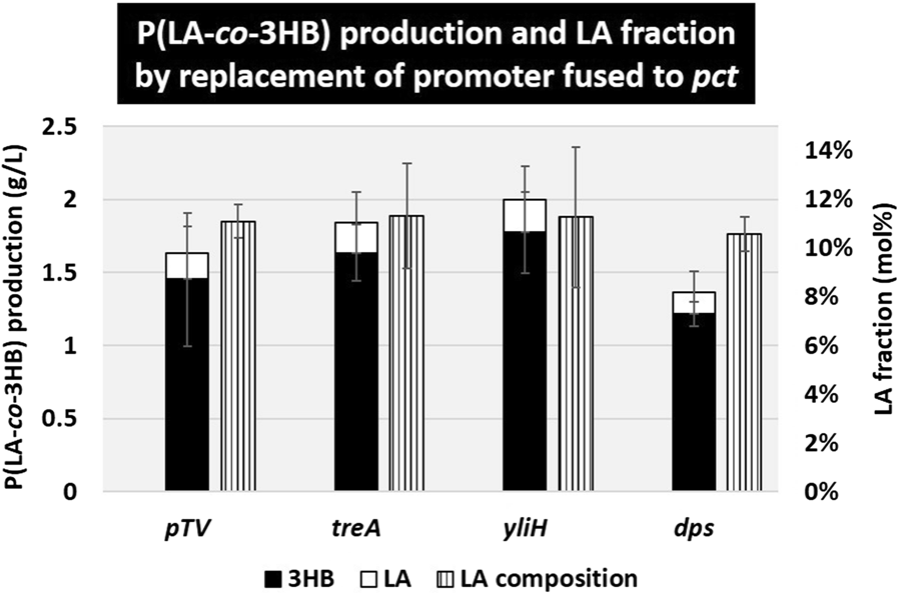
P(LA-*co*-3HB) production by replacement of the promoter fused to *pct*. The bars indicate the amount of 3HB units in the polymer (black), the amount of LA units in the polymer (white), and the LA fraction (vertical stripe). pTV indicates the conventional plasmid pTV118N*pctphaC1*p_s_(ST/QK)*AB.* The gene name indicates the promoter replaced with the *lac* promoter located upstream of *pct*

To increase the LA fraction of P(LA-*co*-3HB), the degree of LA-CoA polymerization in the copolymer needs to be increased. LA is converted from pyruvate by lactate dehydrogenase encoded by *ldhA* located in the *E. coli* genome, which is then converted to LA-CoA by propionyl-CoA transferase (PCT) encoded by *pct* driven by the *lac* promoter in the pTV118N*pctphaC1*p_s_(ST/QK)*AB* plasmid (Fig. 1) [29]. Plasmids were constructed in which the *lac* promoter upstream of *pct* in the plasmid was replaced with a stationary-phase-inducible promoter *dps*, *treA*, or *yliH*, which had a critical effect on the expression of *phaC1STQK-AB* operon. However, the results indicated that there was no significant effect on the amount of P(LA-*co*-3HB) or the LA fraction measured for the transformants harboring these plasmids (Fig. 5). This suggests that PCT activity was not the rate-limiting reaction for LA-CoA production under these experimental conditions.

Next, we cloned *E. coli ldhA* encoding lactate dehydrogenase into a plasmid to control the expression level of LdhA. During this process, we were able to clone the ORF of *ldhA* without a promoter. However, when we attempted to fuse certain promoters to the *ldhA* ORF, we noted nonsense mutations, frameshifts in the *ldhA* ORF sequence, or mutations in the promoter sequence in dozens of the clones obtained (data not shown). Similar results were obtained by supplementing the medium with alanine to alleviate the decrease in the amount of intracellular pyruvate due to lactate production by LdhA [1] as well as by using a buffer solution as the medium to alleviate the effect of lactate production on pH (data not shown). As a result, plasmids for LdhA expression were not obtained. Recently, it was reported that expression of *ldhA* induces persister formation in *E. coli* [32], which may explain why *ldhA* cannot be expressed on a multi-copy plasmid in *E. coli*.

### Improvement of LA fraction in xylose medium using promoter replacement plasmids

**Fig. 6:**
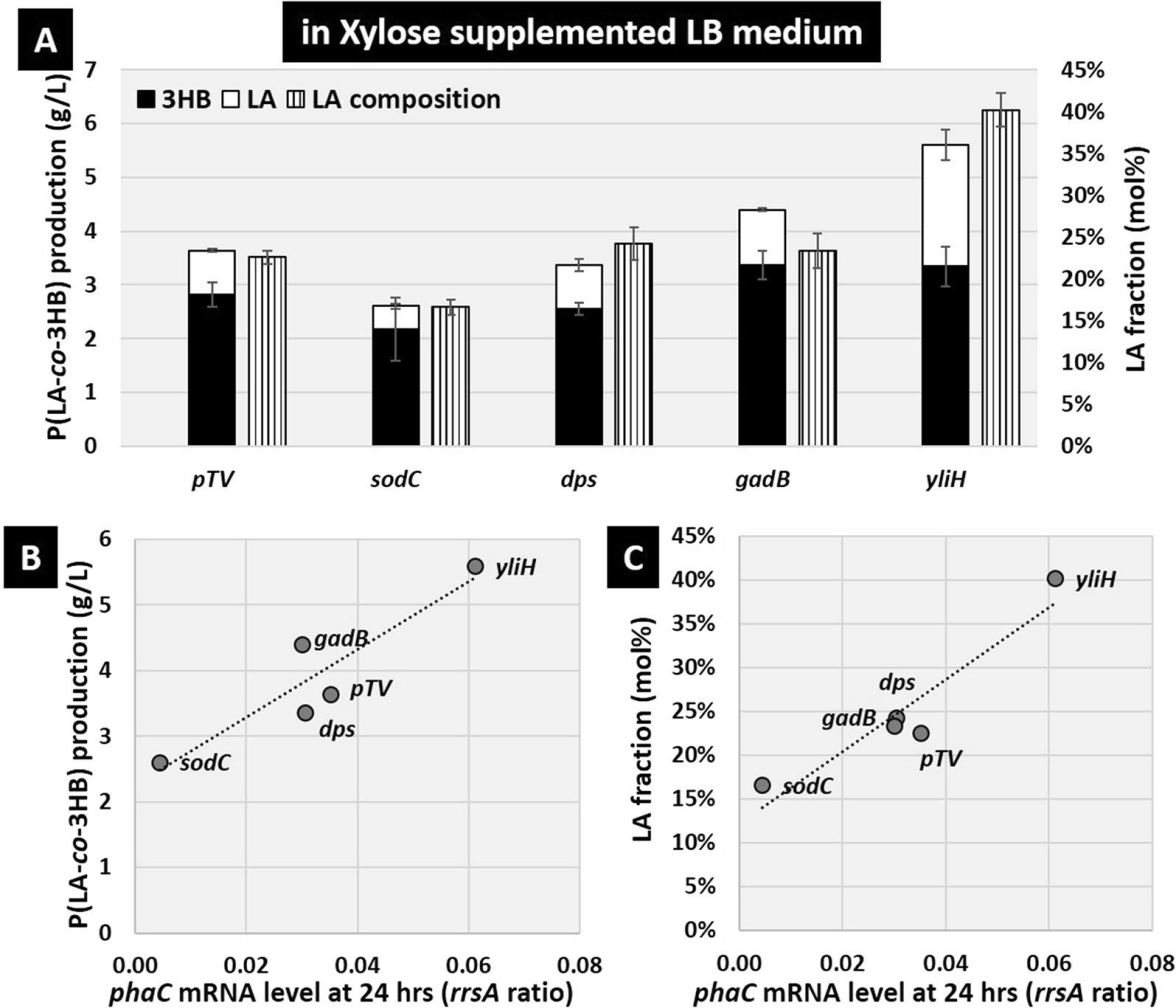
P(LA-*co*-3HB) production and expression level of the *phaC1STQK-AB* operon by replacement of the promoter fused to the *phaC1STQK-AB* operon in xylose-supplemented LB medium. **A** The bars indicate the amount of 3HB units in the polymer (black), the amount of LA units in the polymer (white), and the LA fraction (gray). **B**, **C** The x-axis shows the expression ratio of *phaC* mRNA to the ribosomal RNA, *rrsA*, quantified by RT-qPCR after 24 h of incubation. The y-axis shows the production of P(LA-*co*-3HB) after 48 h of incubation (**B**), and the LA fraction (**C**). pTV indicates the conventional plasmid pTV118N*pctphaC1*p_s_(ST/QK)*AB.* The gene name indicates the promoter replaced with the *phaC* promoter

Previously, Nduko et al. [14] demonstrated that using xylose as a sugar source in the medium led to an increase in the LA fraction of P(LA-*co*-3HB) by suppressing the production of NADPH, thereby lowering the supply of NADPH required for 3HB-CoA synthesis compared to the use of glucose. Next, we measured P(LA-*co*-3HB) production and the LA fraction in xylose-supplemented LB medium using both the conventional plasmid pTV118N*pctphaC1*p_s_(ST/QK)*AB* and stationary-phase-inducible promoter replacement plasmids instead of the *phaC* promoter, in which P(LA-*co*-3HB) production was enhanced in the glucose-supplemented LB medium. In the case of the conventional transformants harboring the pTV118N*pctphaC1*p_s_(ST/QK)*AB*, the P(LA-*co*-3HB) production was 2.7 g/L and the LA fraction was 7.4 mol% in the glucose-supplemented LB medium (Table 1, Additional file 1: Fig. S3), but in the xylose-supplemented LB medium, the production amount was increased slightly to 3.6 g/L and the LA fraction was increased to 22.6 mol% (Fig. 6A, Table 1). In contrast, in transformants harboring the *yliH* promoter replacement plasmid, P(LA-*co*-3HB) production increased to 5.6 g/L and the LA fraction increased markedly to 40.2 mol% (Fig. 6A, Table 1). Transformants harboring the *gadB* promoter replacement plasmid resulted in a slight increase in production to 4.4 g/L, but the LA fraction was 23.4 mol%, the same fraction as with the original plasmid, and the same level or lower for *dps* and *gadB* promoter replacement plasmids. Since these results suggest that these promoter activities differed between glucose- and xylose-supplemented LB media, we quantified the mRNA level of the replaced promoter-dependent *phaC* in this xylose-supplemented LB medium and compared it with the amount and LA fraction of P(LA-*co*-3HB) produced. The results indicate that the P(LA-*co*-3HB) accumulation and LA fraction were correlated with the intracellular *phaC* mRNA level in the stationary phase 24 h after the start of incubation (Fig. 6B, C). At present, there is no knowledge of direct regulation of the *yliH* or *dps* promoters by transcription factors responsive to glucose or xylose in *E. coli*, so the obvious cause for the altered activity of these promoters depending on the sugar type is unknown. It has been reported that LdhA is induced in the stationary phase regardless of the sugar type in *E. coli* [8], suggesting that LA-CoA is synthesized in the stationary phase under this condition. These results indicate that in xylose-supplemented LB medium, both the accumulation of P(LA-*co*-3HB) and the LA fraction depend on the expression of the *phaC1STQK-AB* operon in the stationary phase.

In this study, we aimed to improve P(LA-*co*-3HB) production and alter the LA fraction. We demonstrated the importance of optimizing the expression levels of a set of P(LA-*co*-3HB) synthesis genes in the stationary phase by testing the effects of promoter replacement. In glucose-supplemented LB medium, replacing the *phaC1STQK-AB* promoter of pTV118N*pctphaC1*p_s_(ST/QK)*AB* with the *dps* promoter that showed successfully increased P(LA-*co*-3HB) production threefold to 8.8 g/L. In xylose medium, replacing the *yliH* promoter successfully increased its production by 1.6-fold to 5.6 g/L and increased the LA fraction from 23 to 40 mol%. Thus, it is interesting to note that the same promoter in glucose- and xylose-supplemented LB medium resulted in different effects on P(LA-*co*-3HB) production and the LA fraction, owing to changes in the promoter activity in the stationary phase (Figs. 4 and 6). *E. coli* has approximately 300 different transcriptional regulators, which form a complex hierarchical structure in the cell. As facing the environment changes, each promoter activity changes under the influence of its transcriptional regulatory network [6, 7]. To efficiently produce useful materials using microorganisms, such as P(LA-*co*-3HB), it is important to adapt a suitable gene expression system to fit each condition of the production system used.

## Conclusions

Using a universal model microbial factory of *E. coli*, we have provided proof-of-concept that the replacement of cell growth phase-dependent promoters represents a promising approach for improving the productivity and functional alteration of value-added target products such as P(LA-*co*-3HB) in a microbial factory other than *E. coli*. Notably, this approach synergistically exhibited the beneficial effects in combination with the selection of sugar-based carbon sources. The optimized strain will be subjected to the established high-cell density cultivation [5] for over-production of P(LA-*co*-3HB) to gain a multiple information on polymer properties. In the near future, the polymeric material properties of microbially synthesized P(LA-*co*-3HB) could be systematically altered using advanced synthetic biology based on this approach.

## Materials and methods

### Bacterial strains and medium

*Escherichia coli* K-12 BW25113 [2] was used as a host for polymer production [obtained from the *E. coli* Stock Center (National Bio-Resource Center, Chiba, Japan)]. *E. coli* JM109 cells were used for plasmid amplification and construction.

For polymer production, recombinant *E. coli* harboring pTV118N*pctphaC1*p_s_(ST/QK)*AB* or its derivative plasmids were grown in 20 mL of LB medium containing several different concentrations of glucose or xylose at 30 °C with reciprocal shaking at 180 rpm. Ampicillin (Amp; 100 μg/mL) and kanamycin (25 μg/mL) were added as required. Cell growth, which also reflects P(LA-*co*-3HB) production, was monitored by measuring the turbidity at 600 nm.

### Plasmids construction

The expression vector pTV118N*pctphaC1*p_s_(ST/QK)*AB* [derivative of pTV118N (Takara, Japan), Amp^r^], which harbors genes encoding propionyl-CoA transferase from *Megasphaera elsdenii* (*pct*), engineered polyhydroxyalkanoate (PHA) synthase, termed LPE, with LA-polymerizing activity [*phaC1p*_*s*_(ST/QK)] from *Pseudomonas* sp. 61-3, and 3HB-CoA supplying enzymes β-ketothiolase and acetoacetyl-CoA reductase (*phaA* and *phaB*) from *Cupriavidus necator* (formerly *Ralstonia eutoropha*), was used for P(LA-*co*-3HB) production [29, 30].

Twenty-one *phaC* promoter replacement plasmids were constructed in this study from the original pTV118N*pctphaC1*p_s_(ST/QK)*AB*, with the *Cupriavidus necator*-derived *phaC* promoter replaced with various promoters in *E. coli*. Using the original pTV118N*pctphaC1*p_s_(ST/QK)*AB* as a template, a DNA fragment without the promoter upstream of *phaC* was amplified by PCR using primers 5′-ttattttttcagtcccatgggaccg-3′ and 5′-atgagtaacaagaatagcgatgacttg-3′. The primer sequences with sequences homologous to the vector added at the 5′ end in Additional file 2: Table S1B were then used to amplify the promoter sequences from the *E. coli* genome using the *E. coli* BW25113 genome as the template. The DNA fragments were joined by homologous recombination using the Gibson assembly method to obtain each *phaC* promoter replacement plasmid.

The same procedure was used for the three *pct* promoter replacement plasmids, with the *lac* promoter replaced upstream of *pct*. The plasmid vector fragment was amplified with 5′-atgagaaaagtagaaatcattacagctgaacaag′-3 and 5′-attgcgttgcgctcactg-3′ primers and joined by homologous recombination with the *E. coli* promoter fragment amplified using primers listed in Additional file 2: Table S1C.

*ldhA* from *E. coli* was cloned into pTV118N*pctphaC1*p_s_(ST/QK)*AB* and linked to the *E. coli* promoter using the Gibson assembly method. Vector DNA fragments were amplified using 5′-ccggcatgcaagcttgg-3′ and 5′-atcctatgcccaacaaggcac-3′ primers, and the *ldhA* coding region from the *E. coli* genome was amplified using 5′-atgaaactcgccgtttatagcac-3′ and 5′-ttaaaccagttcgttcgggcag-3′ primers with sequences homologous to the respective promoters added at the 5′ end. The primers listed in Additional file 2: Table S1D were used to amplify the *E. coli* promoter sequence upstream of *ldhA*.

The sequences of the cloned promoters and genes of the constructed plasmids and the sequences of the homologous recombined linkage regions were confirmed by DNA sequencing.

### RT-qPCR analysis

RT-qPCR was performed according to standard procedures [19]. *E. coli* cells were inoculated into M9 minimal medium supplemented with CAA (0.2%) at 37 °C with aeration by constant shaking at 150 rpm. Total RNA was extracted from exponential phase *E. coli* cells (OD_600_ = 0.4) using ISOGEN solution (Nippon Gene, Tokyo, Japan). Total RNA (1 μg) was transcribed into cDNA with random primers using the THUNDERBIRD™ SYBR® qPCR/RT Set (TOYOBO, Osaka, Japan). Quantitative P CR (qPCR) was performed using the THUNDERBIRD™ SYBR® qPCR Mix (TOYOBO) and a LightCycler® 96 system (Roche, Basel, Switzerland). The primer pairs are listed in Additional file 2: Table S1A. The cDNA templates were serially diluted four-fold and used for qPCR analysis. The qPCR mixtures, containing 10 μL of THUNDERBIRD™ SYBR® qPCR Mix (TOYOBO), 1 μL of each primer (5 μM stock), 7 μL of water, and 1 μL of cDNA, were amplified under the following thermal cycling conditions: 2 min at 95 °C, 45 cycles of 10 s at 95 °C and 20 s at 55 °C, and then 20 s at 72 °C. The 16S rRNA expression level was used to normalize the *phaC* mRNA levels of the test samples, and the relative expression levels were quantified using Relative Quantification Software provided by Roche. The results are presented as the averages of three independent experiments.

### Polymer extraction and analyses

Samples from the *E. coli* shake-flask cultures were taken periodically during cultivation and centrifuged at 7500 rpm for 3 min to separate the cells from the medium. The cells were then lyophilized and used for cell growth and polymer analyses. The polymers were analyzed by gas chromatography, as described previously [21]. To determine the cellular polyester content and polymer fraction, approximately 15 mg of dry cells was subjected to methanolysis with a solution consisting of 1.7 mL methanol, 0.3 mL 98% sulfuric acid, and 2.0 mL chloroform at 100 °C for 140 min to convert the constituents to their methyl esters. Addition of 1 mL water to the reaction mixture induced phase separation The lower chloroform layer was used for gas chromatography (GC) on a GL Sciences GC353B FID system equipped with an InertCap-1 capillary column (30 m × 0.25 mm) and a flame ionization detector. Each experiment was repeated at least three times, and the average values are shown.

### Measurement of polymers by Nile-red staining

The Nile-red staining method was performed according to Spiekermann et al. [22]. One milliliter of the cell culture medium was mixed with 10 μL of Nile-red (Sigma-Aldrich Co., St. Louis, MO, USA) dissolved in DMSO (0.4 μg/μL). After vigorous vortexing for 20 s, the mixture was incubated for 5 min at room temperature in the dark. The cells were centrifuged (3 min, 5000×*g*), washed twice with PBS(−) buffer, and the absorbance was measured at 430 nm using a spectrophotometer (V-630BIO, Nihon Bunko, Japan). Each experiment was repeated at least three times, and the average values are shown.

## Supplementary Information

**Additional file 1: Figure S1.** P(LA-*co*-3HB) production and the LA fraction at various glucose concentrations by *E. coli* BW25113 harboring pTV118N*pctphaC1*p_s_(ST/QK)*AB* plasmid in glucose-supplemented LB medium. P(LA-*co*-3HB) production after 48 h of incubation. **Figure S2.** Correlation between P(LA-*co*-3HB) production and the mRNA levels of *phaC* with cultivation time*.* The x-axis shows the expression ratio of *phaC* mRNA to the ribosomal RNA, *rrsA*, quantified by RT-qPCR at 9 (A), 12 (B), 16 (C), and 24 (D) hours of incubation. The y-axis shows the production of P(LA-*co*-3HB) as measured by Nile-red staining of cells after 48 h of incubation. Correlation coefficients between *phaC* mRNA levels and P(LA-*co*-3HB) productions were obtained by the Pearson correlation coefficient and are shown in the lower right corner of each panel. **Figure S3.** P(LA-*co*-3HB) production by replacement of the promoter fused to the *phaC1STQK-AB* operon in glucose-supplemented LB medium. The bars indicate the amount of 3HB units in the polymer (black), the amount of LA units in the polymer (white), and the LA fraction (vertical stripe). pTV indicates the conventional plasmid pTV118N*pctphaC1*p_s_(ST/QK)*AB.* The gene name indicates the promoter replaced with the *phaC* promoter located upstream of the *phaC1STQK-AB* operon. **Figure S4.** P(LA-*co*-3HB) production and the LA fraction at various glucose concentrations by *E. coli* BW25113 harboring pTV118N*pctyliH*p_s_(ST/QK)*AB* plasmid in glucose-supplemented LB medium. P(LA-*co*-3HB) production after 48 h of incubation.

**Additional file 2: Table S1A.** Primer sequences used in RT-qPCR. **Table S1B.** Primer sequences used for construction of phaC promoter replacement plasmid. **Table S1C.** Primer sequences used for construction of pct promoter replacement plasmid. **Table S1D.** Primer sequences used for construction of ldhA promoter replacement plasmid.

## Abbreviations

LA: Lactate
3HB: 3-Hydroxybutyrate
P(LA-*co*-3HB): Poly(LA-*co*-3-hydroxybutyrate)
RT-qPCR: Reverse transcription-quantitative real-time PCR
LB: Luria–Bertani
ORF: Open reading frame
OD: Optical density
PHAs: Polyhydroxyalkanoate
