# Supplementary material for: Cell-growth phase-dependent promoter replacement approach for improved poly(lactate-co-3-hydroxybutyrate) production in Escherichia coli
Source: Microb Cell Fact. 2023 Jul 19;22:131. doi: 10.1186/s12934-023-02143-w (PMC10357597; doi:10.1186/s12934-023-02143-w)
Supplement: Supplementary file 1 — Additional file 1: Figure S1. P(LA-co-3HB) production and the LA fraction at various glucose concentrations by E. coli BW25113 harboring pTV118NpctphaC1ps(ST/QK)AB plasmid in glucose-supplemented LB medium. P(LA-co-3HB) production after 48 h of incubation. Figure S2. Correlation between P(LA-co-3HB) production and the mRNA levels of phaC with cultivation time. The x-axis shows the expression ratio of phaC mRNA to the ribosomal RNA, rrsA, quantified by RT-qPCR at 9 (A), 12 (B), 16 (C), and 24 (D) hours of incubation. The y-axis shows the production of P(LA-co-3HB) as measured by Nile-red staining of cells after 48 h of incubation. Correlation coefficients between phaC mRNA levels and P(LA-co-3HB) productions were obtained by the Pearson correlation coefficient and are shown in the lower right corner of each panel. Figure S3. P(LA-co-3HB) production by replacement of the promoter fused to the phaC1STQK-AB operon in glucose-supplemented LB medium. The bars indicate the amount of 3HB units in the polymer (black), the amount of LA units in the polymer (white), and the LA fraction (vertical stripe). pTV indicates the conventional plasmid pTV118NpctphaC1ps(ST/QK)AB. The gene name indicates the promoter replaced with the phaC promoter located upstream of the phaC1STQK-AB operon. Figure S4. P(LA-co-3HB) production and the LA fraction at various glucose concentrations by E. coli BW25113 harboring pTV118NpctyliHps(ST/QK)AB plasmid in glucose-supplemented LB medium. P(LA-co-3HB) production after 48 h of incubation. [file 12934_2023_2143_MOESM1_ESM.docx]

**Additional file 1**

**
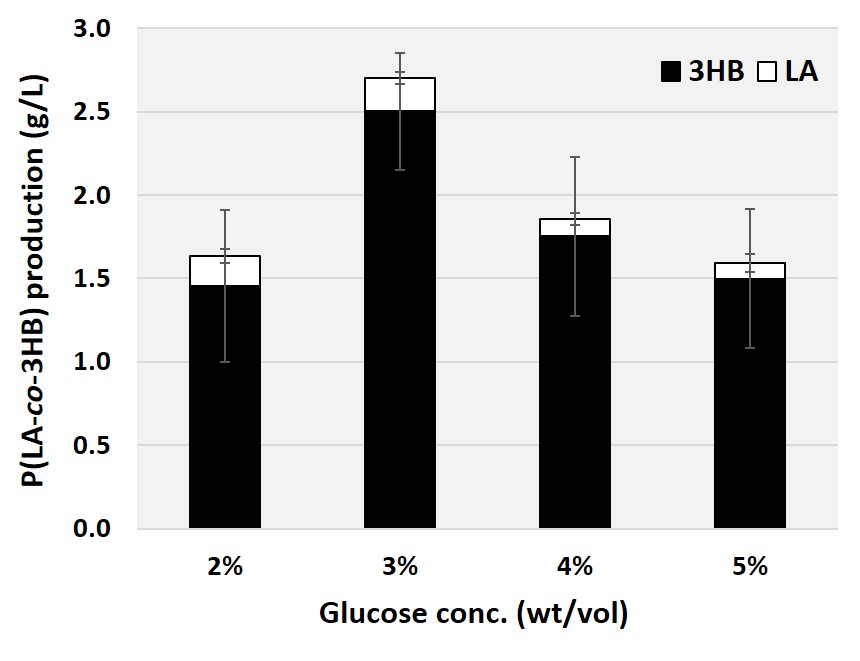
**

**Figure S1. P(LA-*co*-3HB) production and the LA fraction at various glucose concentrations by *E.coli* BW25113 harboring pTV118N*pctphaC1*p_s_(ST/QK)*AB* plasmid in glucose-supplemented LB medium.** P(LA-*co*-3HB) production after 48 hours of incubation.

**
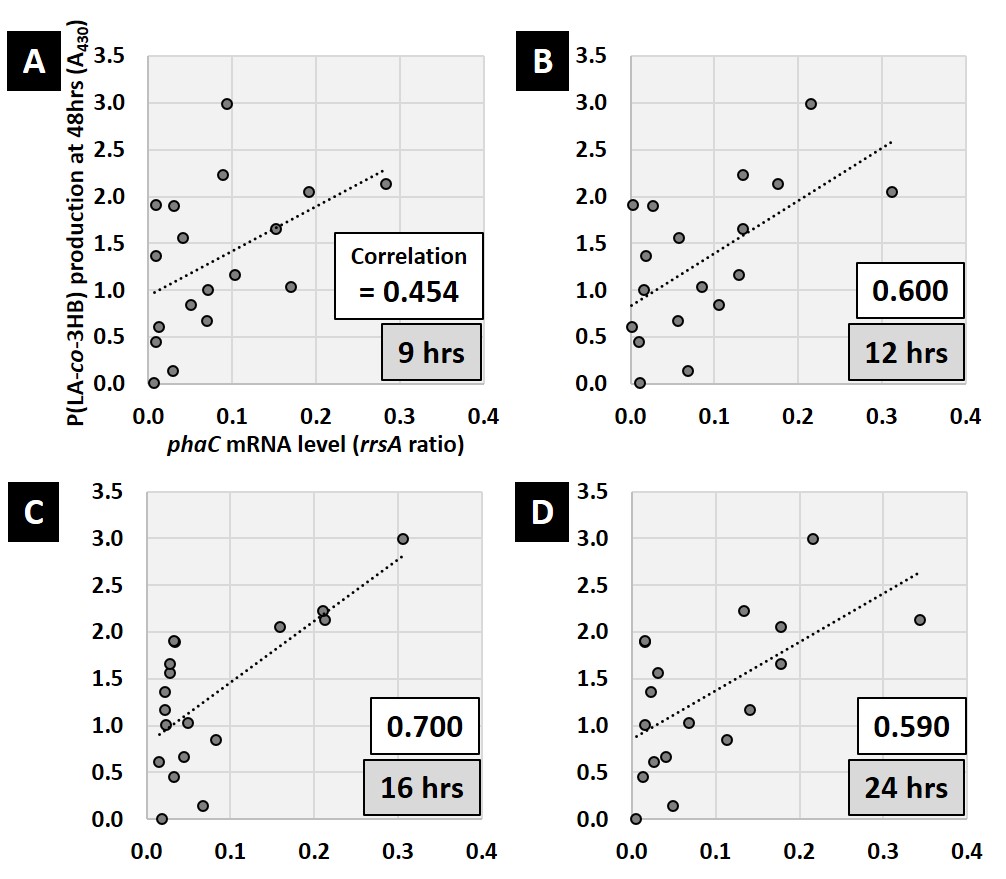
**

**Figure S2. Correlation between P(LA-*co*-3HB) production and the mRNA levels of *phaC* with cultivation time*.*** The x-axis shows the expression ratio of *phaC* mRNA to the ribosomal RNA, *rrsA*, quantified by RT-qPCR at 9 (A), 12 (B), 16 (C), and 24 (D) hours of incubation. The y-axis shows the production of P(LA-*co*-3HB) as measured by Nile-red staining of cells after 48 hours of incubation. Correlation coefficients between *phaC* mRNA levels and P(LA-*co*-3HB) productions were obtained by the Pearson correlation coefficient and are shown in the lower right corner of each panel.

**
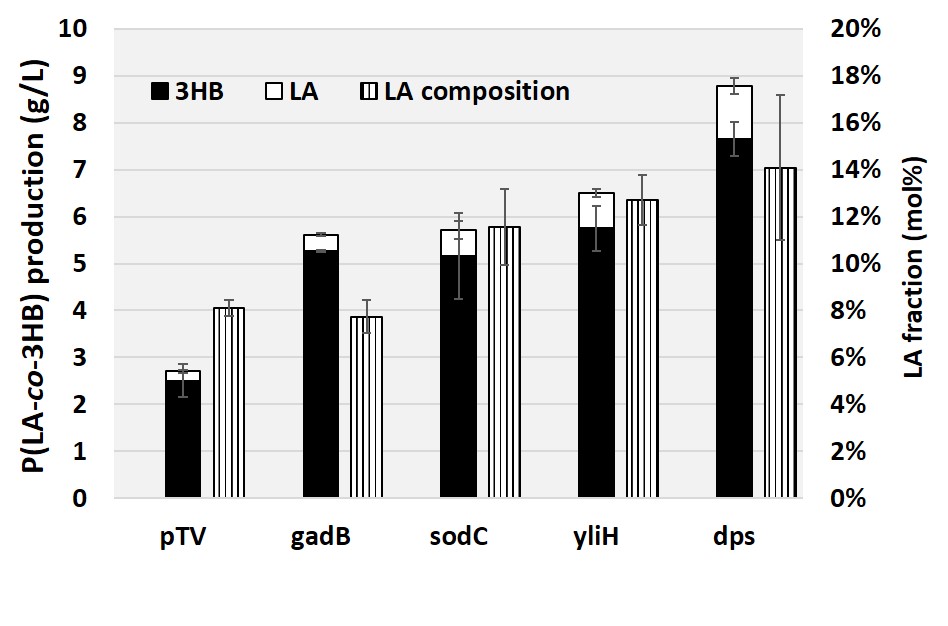
**

**Figure S3. P(LA-*co*-3HB) production by replacement of the promoter fused to the *phaC1STQK-AB* operon in glucose-supplemented LB medium.** The bars indicate the amount of 3HB units in the polymer (black), the amount of LA units in the polymer (white), and the LA fraction (vertical stripe). pTV indicates the conventional plasmid pTV118N*pctphaC1*p_s_(ST/QK)*AB.* The gene name indicates the promoter replaced with the *phaC* promoter located upstream of the *phaC1STQK-AB* operon.

**
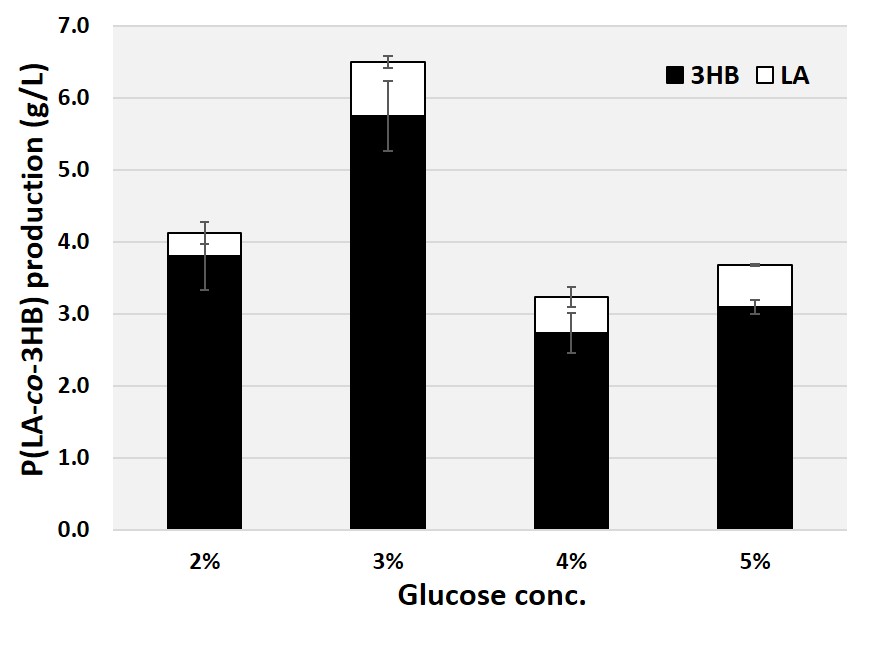
**

**Figure S4. P(LA-*co*-3HB) production and the LA fraction at various glucose concentrations by *E.coli* BW25113 harboring pTV118N*pctyliH*p_s_(ST/QK)*AB* plasmid in glucose-supplemented LB medium.** P(LA-*co*-3HB) production after 48 hours of incubation.
