# Supplementary material for: Cell-growth phase-dependent promoter replacement approach for improved poly(lactate-co-3-hydroxybutyrate) production in Escherichia coli
Source: Microb Cell Fact. 2023 Jul 19;22:131. doi: 10.1186/s12934-023-02143-w (PMC10357597; doi:10.1186/s12934-023-02143-w)
Supplement: Supplementary file 2 — Additional file 2: Table S1A. Primer sequences used in RT-qPCR. Table S1B. Primer sequences used for construction of phaC promoter replacement plasmid. Table S1C. Primer sequences used for construction of pct promoter replacement plasmid. Table S1D. Primer sequences used for construction of ldhA promoter replacement plasmid. [file 12934_2023_2143_MOESM2_ESM.pdf]

Table S1A. Primer sequences used in RT-qPCR

phaC-F	tgcgtggaaaagatctgctg
phaC-F	tcagctcgatgccaaaatgc
rrsA-F	tgcatctgatactggcaagc
rrsA-R	tacgcatttcaccgctacac

Table S1B. Primer sequences used for construction of *phaC* promoter replacement plasmid

csiD phaC F	gggactgaaaaaataacagaagcgatcctcttatgag
csiD phaC R	tattcttgttactcatggagaaccgccgaactatctc
dps phaC F	tgctggccttttgctcctcgctacttttcctctacacc
dps phaC R	attcttgttactcataatttcatatcctcttgatgttatgtc
fic phaC F	gggactgaaaaaataaagtgactcctcagctcttc
fic phaC R	tattcttgttactcatataagatttcccaggtgccgac
gadA phaC F	gggactgaaaaaataaaattgcgtcagaaagtacccgg
gadA phaC R	tattcttgttactcatttcgaactccttaaatttatttgaagg
gadB phaC F	gggactgaaaaaataagccaaaataataacagccccgtc
gadB phaC R	tattcttgttactcattttaaactccttaaaatgattggatcg
glpD phaC	attcttgttactcatgctgccctcattcactttc
glpD phaC F	tgctggccttttgctctatagaccacatagtagccctg
modA phaC F	tgctggccttttgctcaaaacgaacgccacgcaaagc
modA phaC R	attcttgttactcattggtaacccccttaatgtaacg
osmB phaC F	gggactgaaaaaataagaaaaaaagcgaaagtatggttggcc
osmB phaC R	tattcttgttactcataatactctctcctgaatttatgattcac
osmC phaC F	gggactgaaaaaataatgttgctctcctgtgggc
osmC phaC R	tattcttgttactcataacatagtgattctccgtgtctgtg
osmY phaC F	gggactgaaaaaataacgatttattcctgtatgtttgctc
osmY phaC R	tattcttgttactcattaacctcgcttacatcgctacc
poxB phaC F	gggactgaaaaaataaccggcacgagaattttacgcc
poxB phaC R	tattcttgttactcatggttctccatctcctgaatg
slp phaC F	gggactgaaaaaataagttactatccttatcaacaaatattaaaac
slp phaC R	tattcttgttactcatctttacgacactctcccgatac
sodC phaC F	gggactgaaaaaataactcgaccaactacaacaactgc
sodC phaC R	tattcttgttactcataggacctccgttcattgtg
treA phaC F	tgctggccttttgctcgcgatacaaccagaagaaacgc
treA phaC R	attcttgttactcatcaatcattctcctttggcgaaac
uspB phaC F	gggactgaaaaaataaccggtgtataggtcagagtag
uspB phaC R	tattcttgttactcatacctcctccccggcgacc
uxuA phaC R	attcttgttactcatgatttttcctctttcacatagtgg
uxuA phaCF	tgctggccttttgctcctggttgaaccgttgttattttggg
wrbA phaC F	gggactgaaaaaataattttgccgtgactgctcttaccac
wrbA phaC R	tattcttgttactcatttctaaccactcctcgtgttatc
yceP phaC F	gggactgaaaaaataagaagtcaccatagcgaaaacttctc
yceP phaC R	tattcttgttactcatcatggccccctaattcgtg
yciF phaC F	gggactgaaaaaataaacgtcatttttccgcccaacag
yciF phaC R	tattcttgttactcatatttttctccagtgaaatcactgc
yjbJ phaC F	gggactgaaaaaataaaatcaagacctcatcgttagg
yjbJ phaC R	tattcttgttactcatgtcggggatcgtagcgttac
yliH phaC F	tgctggccttttgctcgtgatttccagcactttcggg
yliH phaC R	attcttgttactcatacttcgatcctcctcttcc

Table S1C. Primer sequences used for construction of *pct* promoter replacement plasmid

dps pct F	gtgagcgcaacgcaatctcgctacttttcctctacacc
dps pct R	tttctacttttctcataatttcatatcctcttgatgttatgtc
treA pct F	gtgagcgcaacgcaatgcgatacaaccagaagaaacgc
treA pct R	attcttgttactcatcaatcattctcctttggcgaaac
yliH pct F	gtgagcgcaacgcaatgtgatttccagcactttcg
yliH pct R	tttctacttttctcatacttcgatcctcctcttcc

Table S1D. Primer sequences used for construction of *ldhA* promoter replacement plasmid

dps ldhA F	caagcttgcatgccggctcgctacttttcctctacacc
dps ldhA R	aaacggcgagtttcataatttcatatcctcttgatgttatgtc
treA ldhA F	caagcttgcatgccgggcgatacaaccagaagaaacgc
treA ldhA R	aaacggcgagtttcatcaatcattctcctttggcgaaac
yliH ldhA F	caagcttgcatgccgggtgatttccagcactttcggg
yliH ldhA R	aaacggcgagtttcatacttcgatcctcctcttcc
